# Mechanical valve replacement without anticoagulation: a case report

**DOI:** 10.1093/ehjcr/ytaa566

**Published:** 2021-01-15

**Authors:** Yapeng Wang, Min Lin, Shenglin Ge, Junbo Feng

**Affiliations:** Department of Cardiovascular Surgery, The First Affiliated Hospital of Anhui Medical University, 218 Jixi Road, Hefei 230022, Anhui, People’s Republic of China

**Keywords:** Case report, Valve replacement, Anticoagulation, Factor X deficiency

## Abstract

**Background:**

For patients who undergo mechanical valve replacement, the greatest disadvantage is that they require long-term or permanent use of anticoagulant therapy to prevent thromboembolism. To date, mechanical valve replacement without anticoagulation has been published in the literature.

**Case summary:**

We present the case of a 75-year-old female who underwent mechanical mitral valve replacement (MVR) on mid**-**June, 2007. However, this patient had not been taking anticoagulant medication since she experienced warfarin overdose in the first month after the operation. She had been well without using any anticoagulation, and there were no complications of the mechanical valve.

**Discussion:**

There was no thrombosis for such a long period of time because she suffered from FX deficiency. To the best of our knowledge, she may be the only patient who has been well without any anticoagulation since not taking warfarin 12 years ago.

Learning points

- The patient suffers Factor X (FX) deficiency.
- To the best of our knowledge, she may be the only patient who has been well without any anticoagulation since not taking warfarin 12 years ago.

## Introduction

Prosthetic heart valve replacement is recommended for patients with severe cardiac valve disease and is performed in many patients worldwide every year.^1^ Mechanical valves are more durable than bioprosthetic valves,^2^ but patients with these valves require lifelong anticoagulant therapy. Warfarin provide excellent protection against thrombo-embolic complications in patients with mechanical heart valves,^3^ but these patients require lifelong monitoring of coagulation studies. Because excessive or insufficient anti**-**coagulant effects may cause severe clinical symptoms such as bleeding and thrombo-embolic events, it is difficult for clinicians to estimate the optimal initial dosage of warfarin to attain such a narrow therapeutic international normalized ratio (INR) range for every patient.

## Timeline

**Table:** 

Dates	Presentation	Investigations	Findings
14 June 2007	Progressive dyspnoea	Echocardiography	Severe mitral regurgitation
July 2007 July 2007 to October 2019 October 2019	Skin purpura Asymptomatic Oedema of the legs	Monitor anticoagulant function Telephone and outpatient follow-up Echocardiography	Excessive anticoagulation without bleeding She was well without any anticoagulation Right ventricular dysfunction

## Case presentation

A 75-year-old Chinese woman was admitted to the hospital with symptoms of progressive oedema of the lower limbs. On physical exam, her head exam revealed a normocephalic, atraumatic head with no palpable or visible masses. A neck exam revealed no lymphadenopathy, jugular venous distention, or carotid bruits. A cardiovascular exam was significant for abnormal S1 and S2 but no murmurs or thrills on auscultation. Breath sounds were clear and symmetric bilaterally, without any crackles, wheezes, or rhonchi. Her abdomen was soft, non-distended, and non-tender, with normal bowel sounds and no organomegaly.

The patient underwent mechanical mitral valve (GK-3 tilting disc, 27 mm, made in China) replacement surgery on mid**-**June, 2007. She had a history of hypertension for 15 years. At this time, she was admitted to our hospital again because of right ventricular dysfunction. The patient was discharged after symptoms of shortness of breath, leg swelling had been improved with diuretic therapy.

We found that the patient had been taking 0.625 mg warfarin per day since one month after surgery, but due to excessive anticoagulation, she suffered from skin purpura without bleeding and neurological complications. Therefore, she had stopped taking warfarin and other anticoagulant treatments since she experienced excessive anticoagulation. Interestingly, her INR had always remained in the range of 2.0–2.5 until now without any anticoagulants. Follow-up interval for her will be half a year in the future.

**Figure 1:**
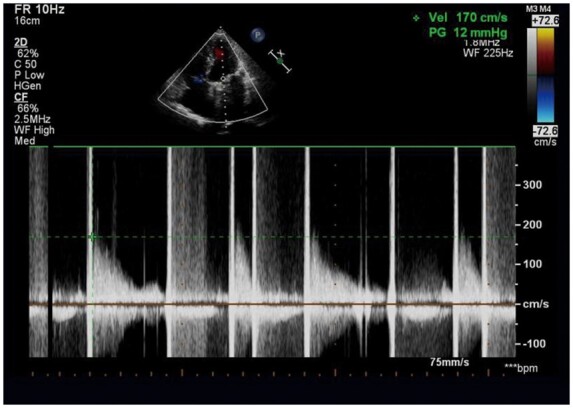
Transthoracic echocardiography showing a normal**-**functioning mechanical mitral prosthesis.

**Figure 2:**
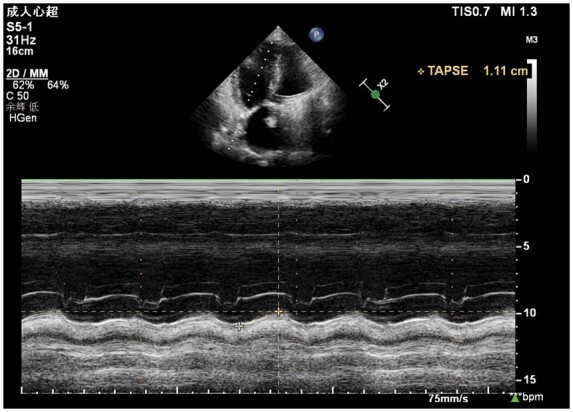
Transthoracic echocardiography showing right ventricular dysfunction.

**Figure 3:**
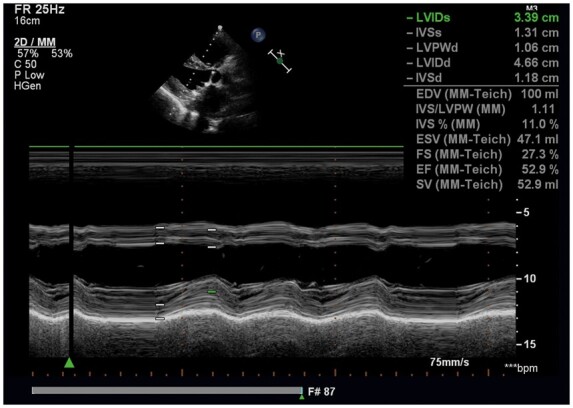
Transthoracic echocardiography showing normal left ventricular function.

Repeat echocardiography showed a normal-functioning mechanical mitral prosthesis (*Figure 1*). On 29 September 2019, transthoracic echocardiography showed right ventricular enlargement (*Figure 2*) and normal left ventricular function (*Figure 3*), and the mitral valve was working well without any vegetation (*Videos 1 and 2*). There was no thrombosis in the heart valve or left atrium. The preoperative PT and INR of this patient were 22.5 s and 2.02, respectively. Warfarin-related genes were tested (*Table 1*). These genes were normal. We found that the patient suffered from factor X (FX) deficiency (*Table 2*). Therefore, we considered that this patient, without any anticoagulants, did not have thrombo-embolic complications due to the FX deficiency.

**Table 1:** Gene studies of the patient

Gene	Genotype	Result
MTHFR (C677T)	CC, CT, TT	CC
PAI-1 (5G/4G)	5G/5G, 5G/4G, 4G/4G	5G/5G
CYP2C9*2 (R144C, C→T)	CC, CT, TT	CC
CYP2C9*3 (I359L, A→C)	AA, AC, CC	AA
VKORC1 (G-1639A)	GG, GA, AA	AA

**Table 2:** Coagulation factor studies of the patient

Factor	Result	Normal
II: C	76.40% ↓	79–131%
V: C	105.10%	62–139%
VII: C	75.30%	50–129%
VIII: C	126.70%	50–150%
IX: C	113.90%	65–150%
X: C	7.10% ↓↓	77–131%
XI: C	83.70%	65–150%
XII: C	71.80%	50–150%
PT	24.60 s ↑	9.9–12.8 s
APTT	48.00 s ↑	25.1–36.5 s
INR	2.22	

## Discussion

Cardiac valve replacement is one of the most effective methods for the treatment of mid- to late-stage cardiac valvular diseases. In China, cardiac valve replacements account for 30% of cardiac procedures.^4^ With the continuous improvements in perioperative management and surgical techniques, heart valve surgery has lower mortality. However, there is a high incidence of thrombo-embolic events of approximately 1–4% per year.^5^ The bleeding risk is significant, ranging from 2% to 9% per year.^6^ Therefore, the greatest disadvantage of this surgery is that patients require long-term or permanent use of anticoagulant therapy to prevent thrombo-embolic events.

Warfarin is an effective drug for addressing this problem but increases the risk of major bleeding at the same time.^7^ Warfarin interferes with the hepatic synthesis of vitamin K-dependent clotting factors II, VII, IX, and X, resulting in their eventual depletion and a prolongation in the clotting time, as measured by the PT and INR. Compared with other drugs, warfarin has been viewed as the most frequently used clinical oral anticoagulant drug due to its relatively low cost.^8^^,^^9^ However, the toxic dose of warfarin is close to the dosage required to achieve a pharmaceutical effect. The warfarin dosage response is related to demographic, environmental, clinical and, especially, genetic factors.^10^ Due to the narrow therapeutic range as well as interactions and genetic variants, patients who experience warfarin overdose need genetic testing for the initial estimate of warfarin dose and the close monitoring of the intensity of anticoagulation with warfarin.^11^ In our hospital, we have been able to widely perform warfarin-related gene testing in patients with abnormal coagulation function. If a patient with a mechanical heart valve presents with warfarin overdose, vitamin K and fresh-frozen plasma should be given. The American College of Chest Physicians (2008) guidelines recommend oral doses of 1–2.5 mg vitamin K for an INR between 5 and 9 and 2.5–5 mg for all patients with an INR ≥ 9 but with no significant bleeding.^12^ The INR is then monitored every 4–6 h after administering vitamin K. When the INR is <3, the lowest dose of warfarin is given to prevent thrombosis.^12^

Factor X, a vitamin K-dependent plasma glycoprotein, plays a pivotal role in the coagulation cascade. Factor X is the first enzyme in the common pathway of thrombin formation. Factor X deficiency is a rare, recessively inherited bleeding disorder representing 10% of all rare bleeding diseases and affecting 1 in every 1 000 000 people.^13^ Factor X deficiency can be congenital or acquired.^14^ The diagnosis of factor X deficiency is usually suspected when both the prothrombin time and activated partial thromboplastin time are abnormal and are corrected upon mixing 1:1 with normal plasma.^15^ The functional activity of Factor X (FX: C) is quantified by performing a prothrombin time-based assay using rabbit thromboplastin and factor X-deficient plasma. Accordingly, patients are classified into three groups: severe (FX: C, <1%), moderate (FX: C, 1–4%), and mild (FX: C, 6–10%).^16^

Rivaroxaban is a direct and selective coagulation factor Xa inhibitor. Indications for the use of these agents include the prevention of stroke in non-valvular atrial fibrillation and for the prevention and treatment of deep vein thrombosis and pulmonary embolism as well as the prevention of venous thrombosis after orthopaedic surgery, but these agents are not indicated for patients undergoing cardiac valve replacement.^17^ There was a case report where a patient who underwent mechanical aortic valve replacement was administered the oral anticoagulant rivaroxaban instead of warfarin, which caused a significant gradient and thrombosis on one leaflet of the valve.^18^ This patient died due to complications from the thrombotic valve. However, for this patient with FX deficiency, she had not been taking any anticoagulants and did not have any complications due to the mechanical valve.

## Conclusion

We believe that there was no thrombosis for such a long period of time because she suffered from FX deficiency. We have not found out other mechanisms to explain this phenomenon so far.

## Lead author biography

**Figure:**
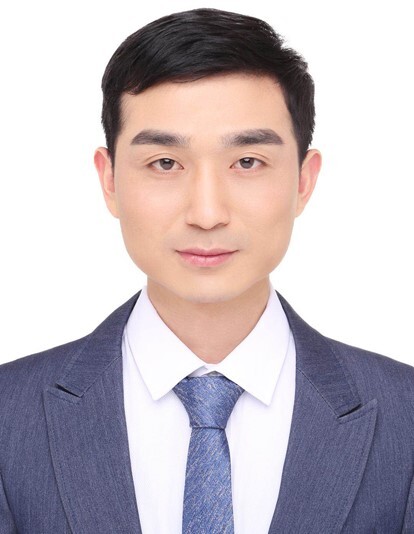


I am a cardiac surgeon and have two English papers.

## Supplementary material

Supplementary material is available at *European Heart Journal - Case Reports* online.

**Slide sets:** A fully edited slide set detailing these cases and suitable for local presentation is available online as Supplementary data. 

**Consent:** The authors confirm that written consent for submission and publication of this case report including images and associated text has been obtained from the patient in line with COPE guidance.

**Conflict of interest:** None declared.

**Funding:** None declared. 

## Supplementary Material

ytaa566_Supplementary_Data
