# Supplementary material for: Mechanical valve replacement without anticoagulation: a case report
Source: Eur Heart J Case Rep. 2021 Jan 15;5(1):ytaa566. doi: 10.1093/ehjcr/ytaa566 (PMC7898660; doi:10.1093/ehjcr/ytaa566)
Supplement: ytaa566_Supplementary_Data [file ytaa566_supplementary_data.zip › A case report.pptx]

## Slide 1
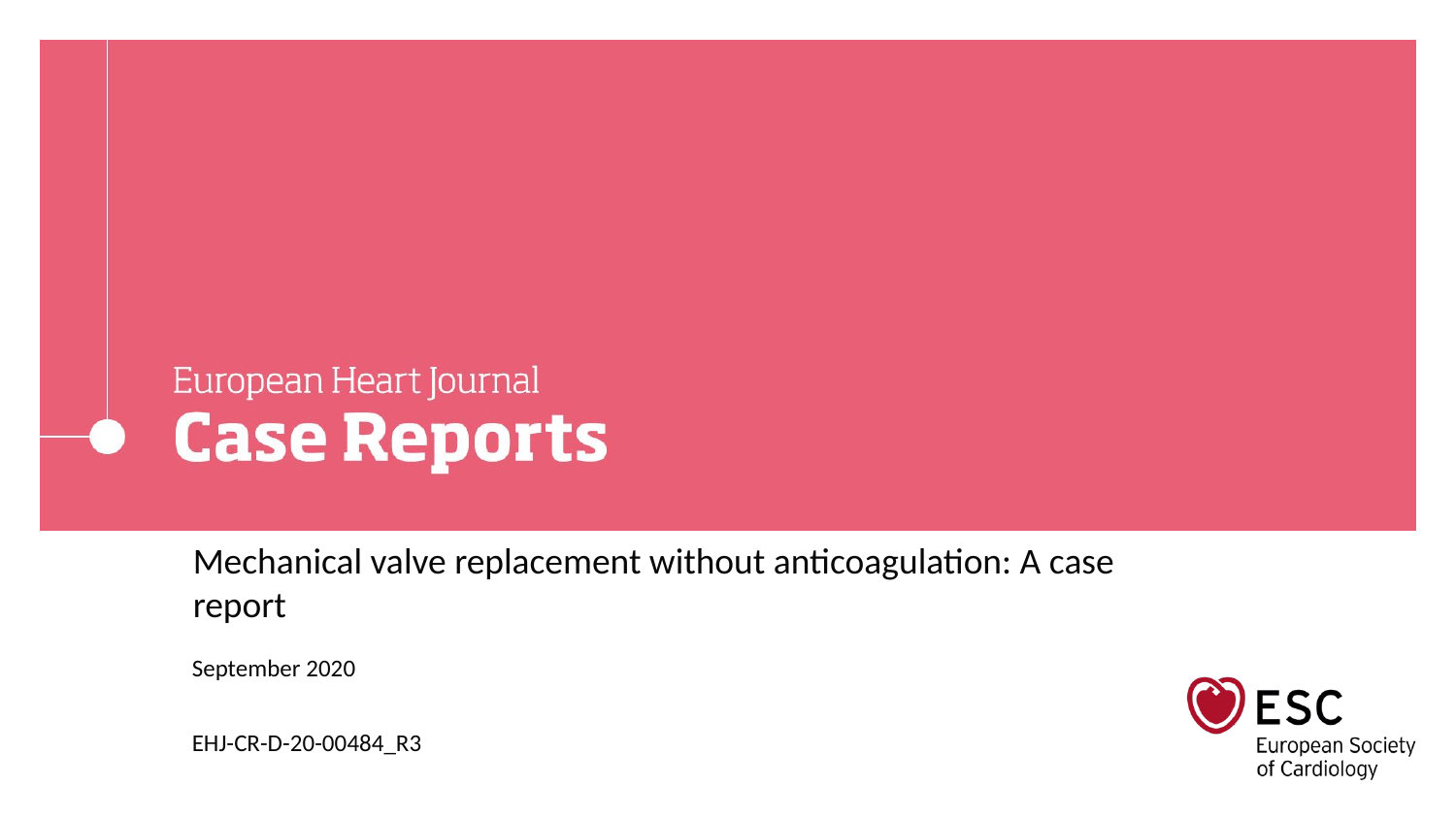

# Mechanical valve replacement without anticoagulation: A case report
September 2020
EHJ-CR-D-20-00484_R3

## Slide 2
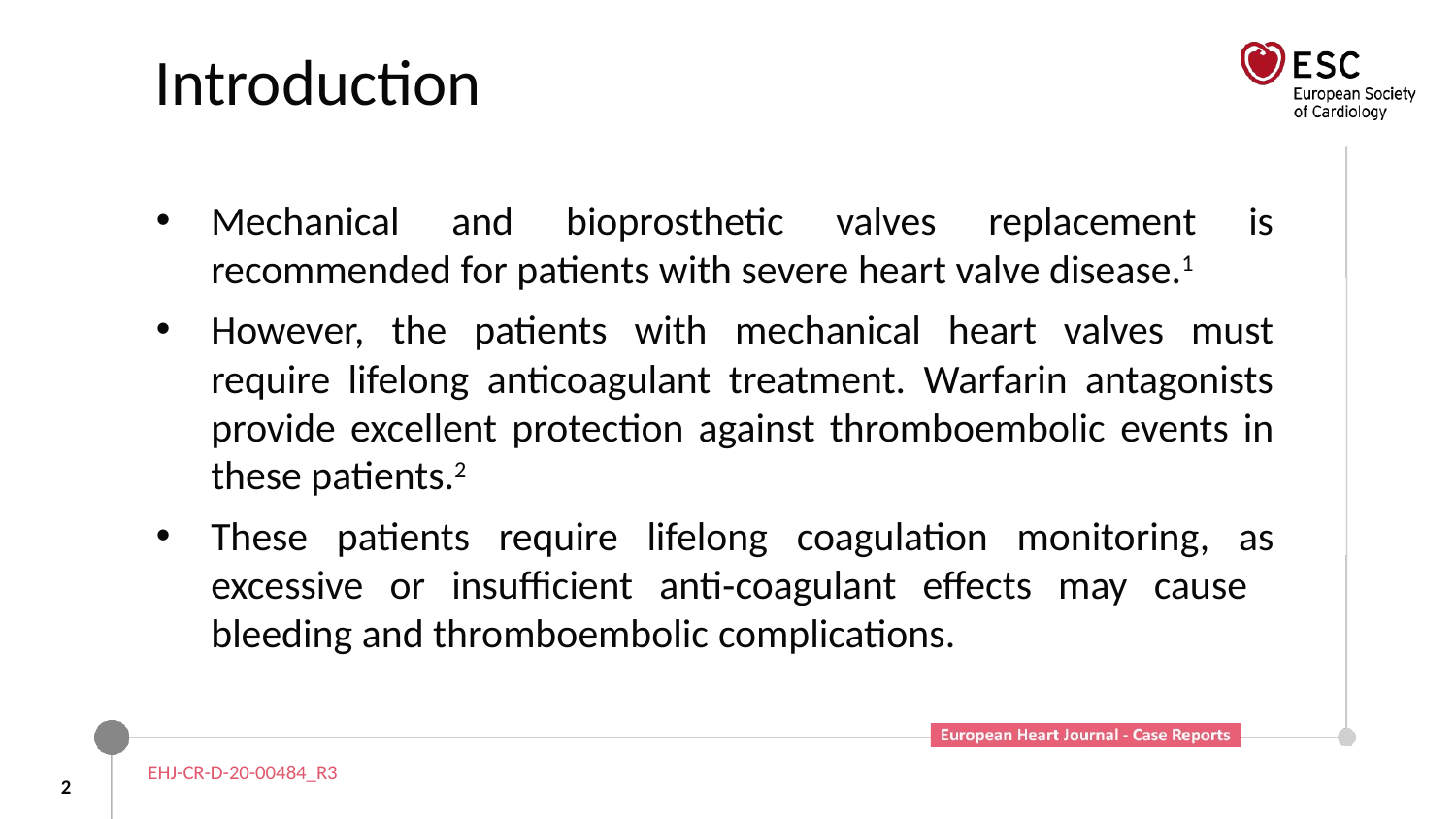

# Introduction
Mechanical and bioprosthetic valves replacement is recommended for patients with severe heart valve disease.1
However, the patients with mechanical heart valves must require lifelong anticoagulant treatment. Warfarin antagonists provide excellent protection against thromboembolic events in these patients.2
These patients require lifelong coagulation monitoring, as excessive or insufficient anti‑coagulant effects may cause bleeding and thromboembolic complications.
EHJ-CR-D-20-00484_R3
2

## Slide 3
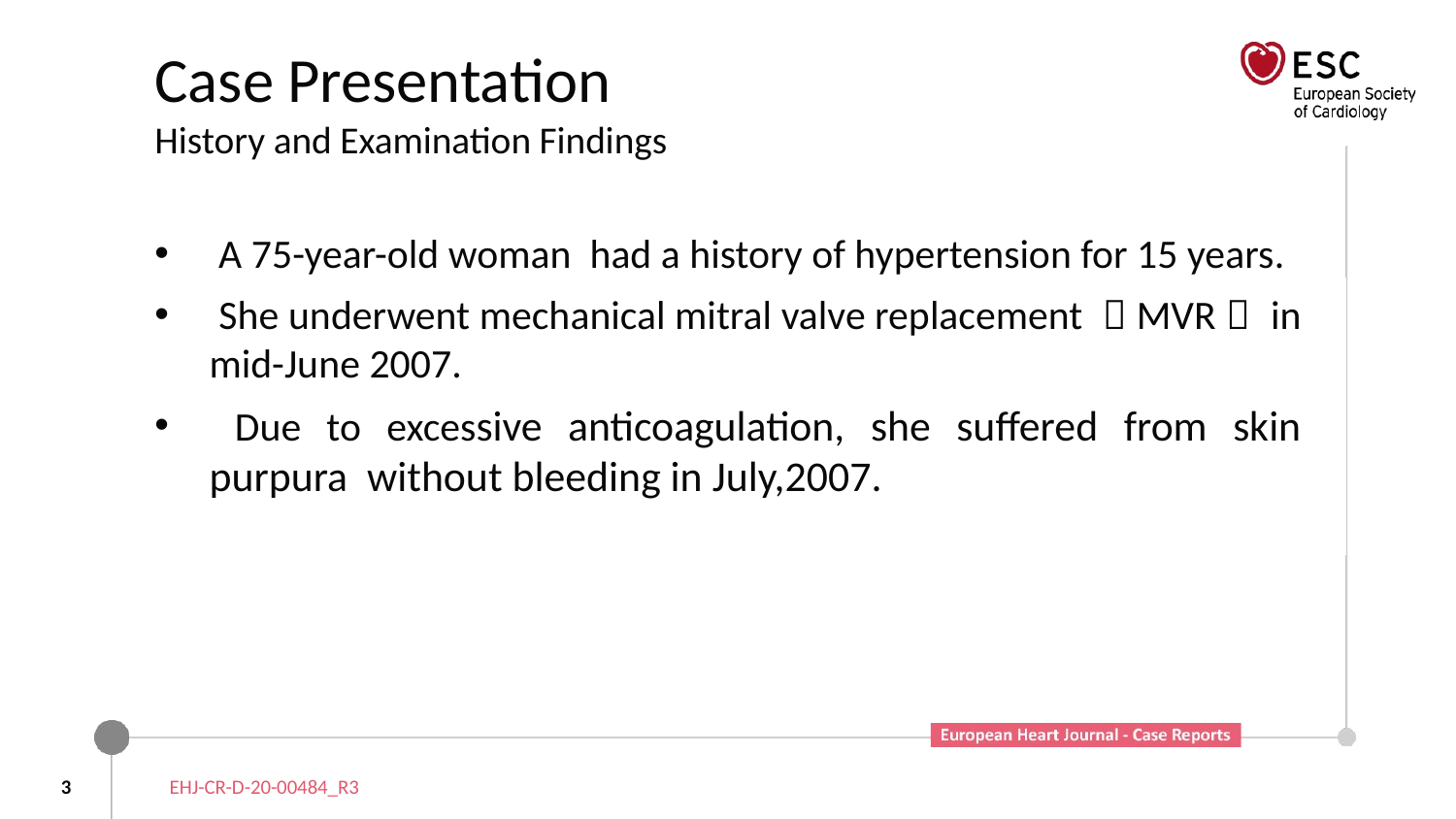

# Case Presentation History and Examination Findings
 A 75-year-old woman had a history of hypertension for 15 years.
 She underwent mechanical mitral valve replacement （MVR） in mid-June 2007.
 Due to excessive anticoagulation, she suffered from skin purpura without bleeding in July,2007.
3
EHJ-CR-D-20-00484_R3

## Slide 4
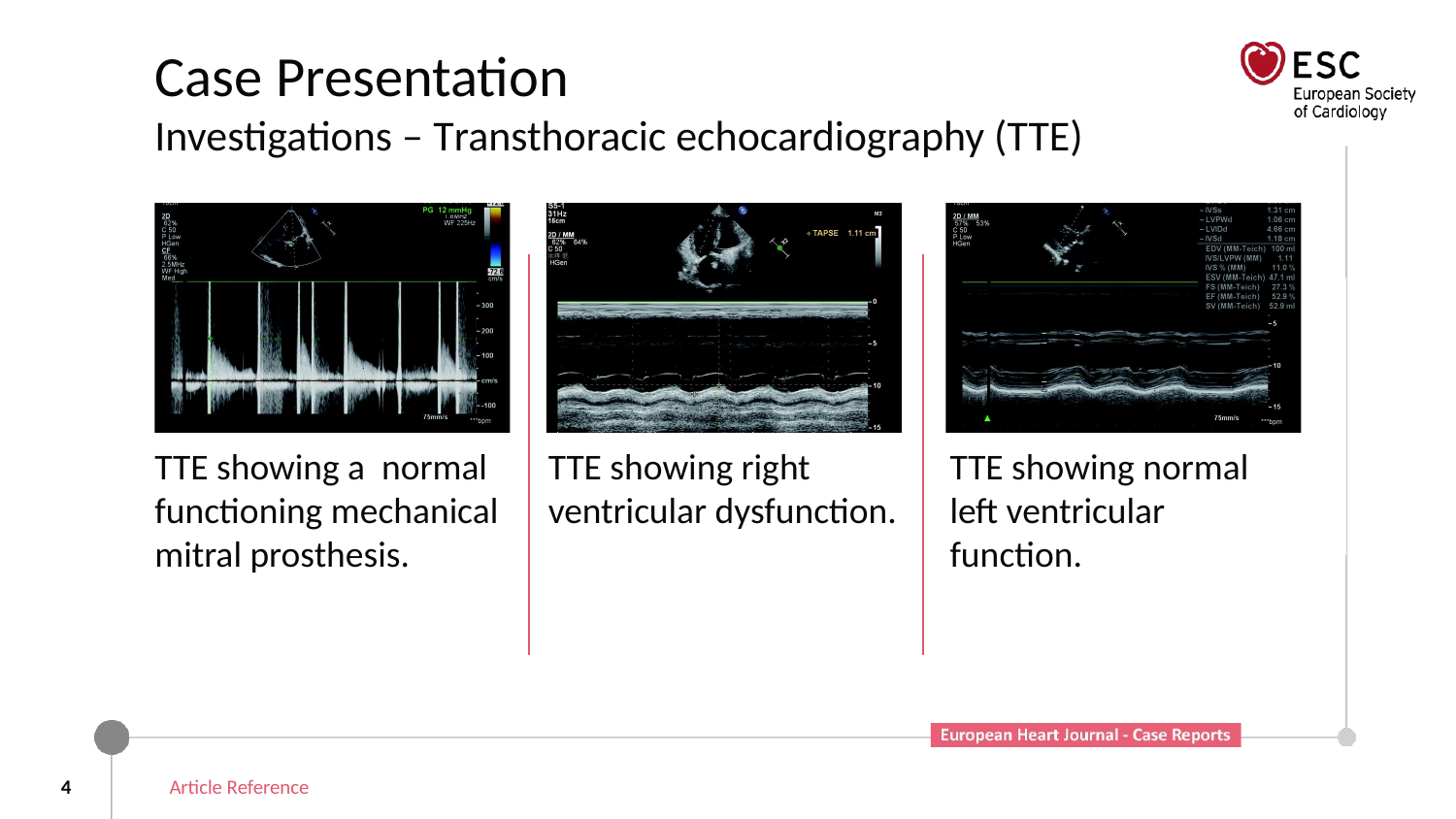

# Case Presentation Investigations – Transthoracic echocardiography (TTE)
TTE showing a normal functioning mechanical mitral prosthesis.
TTE showing right ventricular dysfunction.
TTE showing normal left ventricular function.
4
Article Reference

## Slide 5
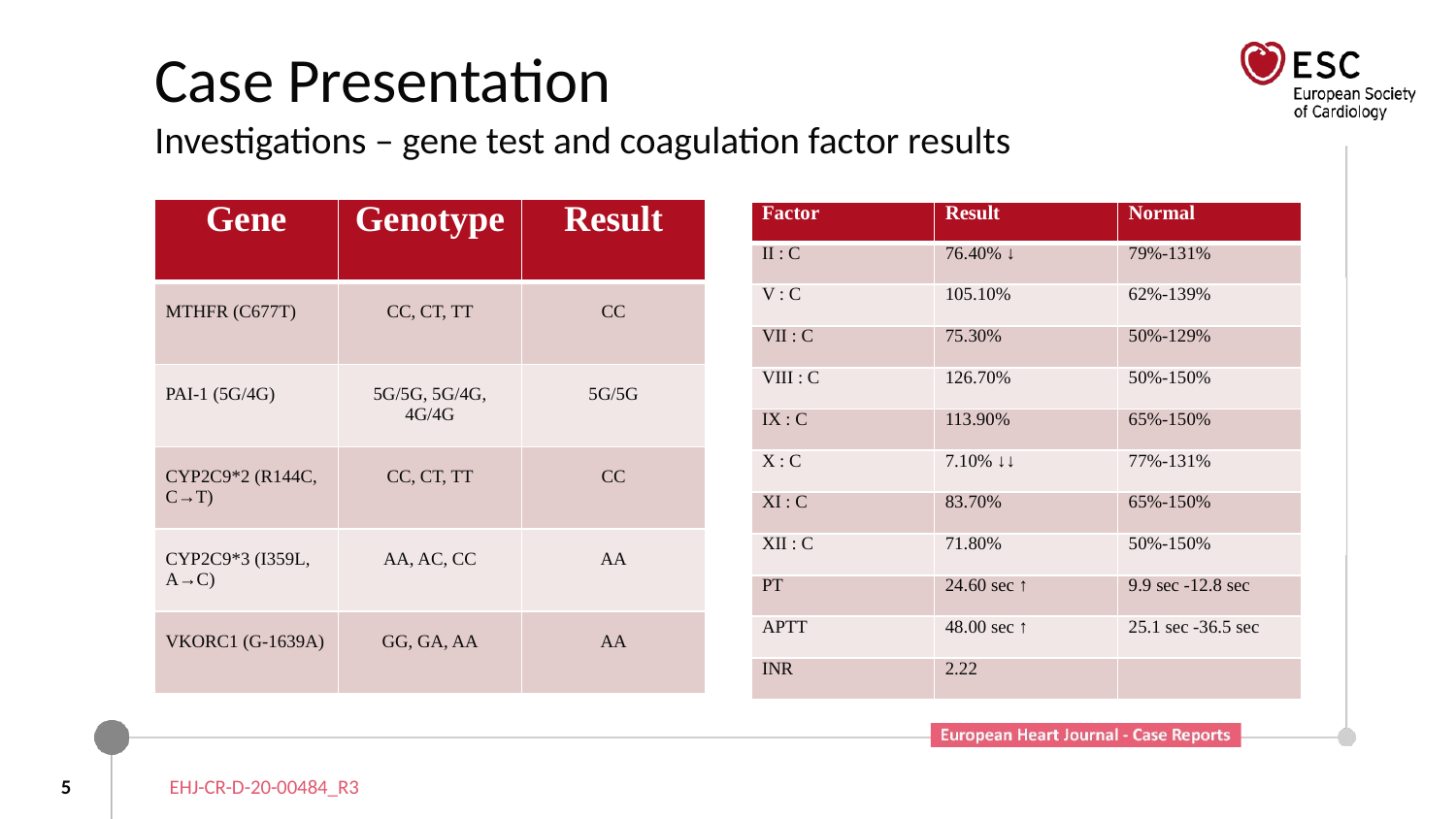

# Case Presentation Investigations – gene test and coagulation factor results
| Gene | Genotype | Result |
| --- | --- | --- |
| MTHFR (C677T) | CC, CT, TT | CC |
| PAI-1 (5G/4G) | 5G/5G, 5G/4G, 4G/4G | 5G/5G |
| CYP2C9\*2 (R144C, C→T) | CC, CT, TT | CC |
| CYP2C9\*3 (I359L, A→C) | AA, AC, CC | AA |
| VKORC1 (G-1639A) | GG, GA, AA | AA |
| Factor | Result | Normal |
| --- | --- | --- |
| II : C | 76.40% ↓ | 79%-131% |
| V : C | 105.10% | 62%-139% |
| VII : C | 75.30% | 50%-129% |
| VIII : C | 126.70% | 50%-150% |
| IX : C | 113.90% | 65%-150% |
| X : C | 7.10% ↓↓ | 77%-131% |
| XI : C | 83.70% | 65%-150% |
| XII : C | 71.80% | 50%-150% |
| PT | 24.60 sec ↑ | 9.9 sec -12.8 sec |
| APTT | 48.00 sec ↑ | 25.1 sec -36.5 sec |
| INR | 2.22 | |
5
EHJ-CR-D-20-00484_R3

## Slide 6
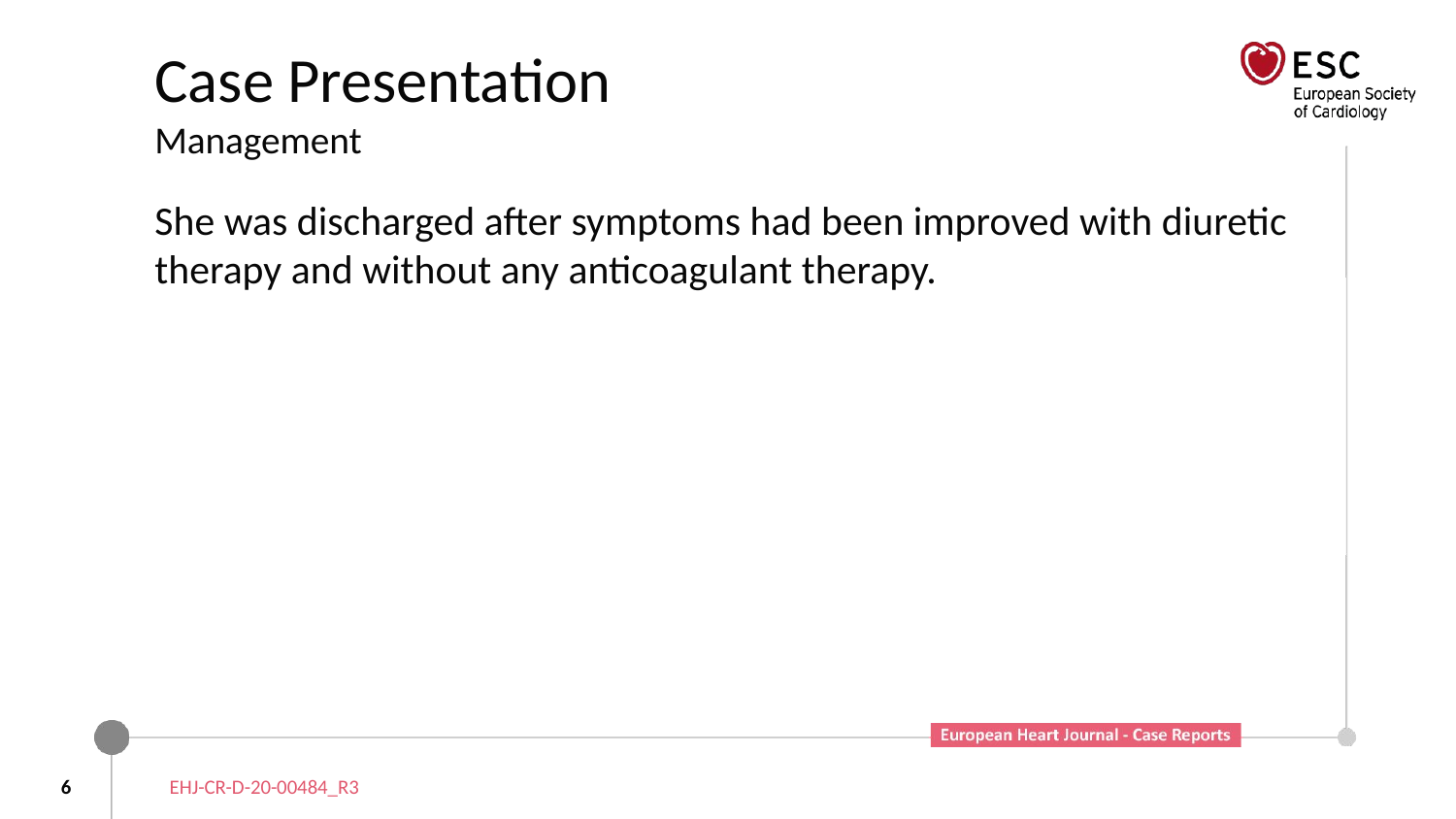

# Case Presentation Management
She was discharged after symptoms had been improved with diuretic therapy and without any anticoagulant therapy.
6
EHJ-CR-D-20-00484_R3

## Slide 7
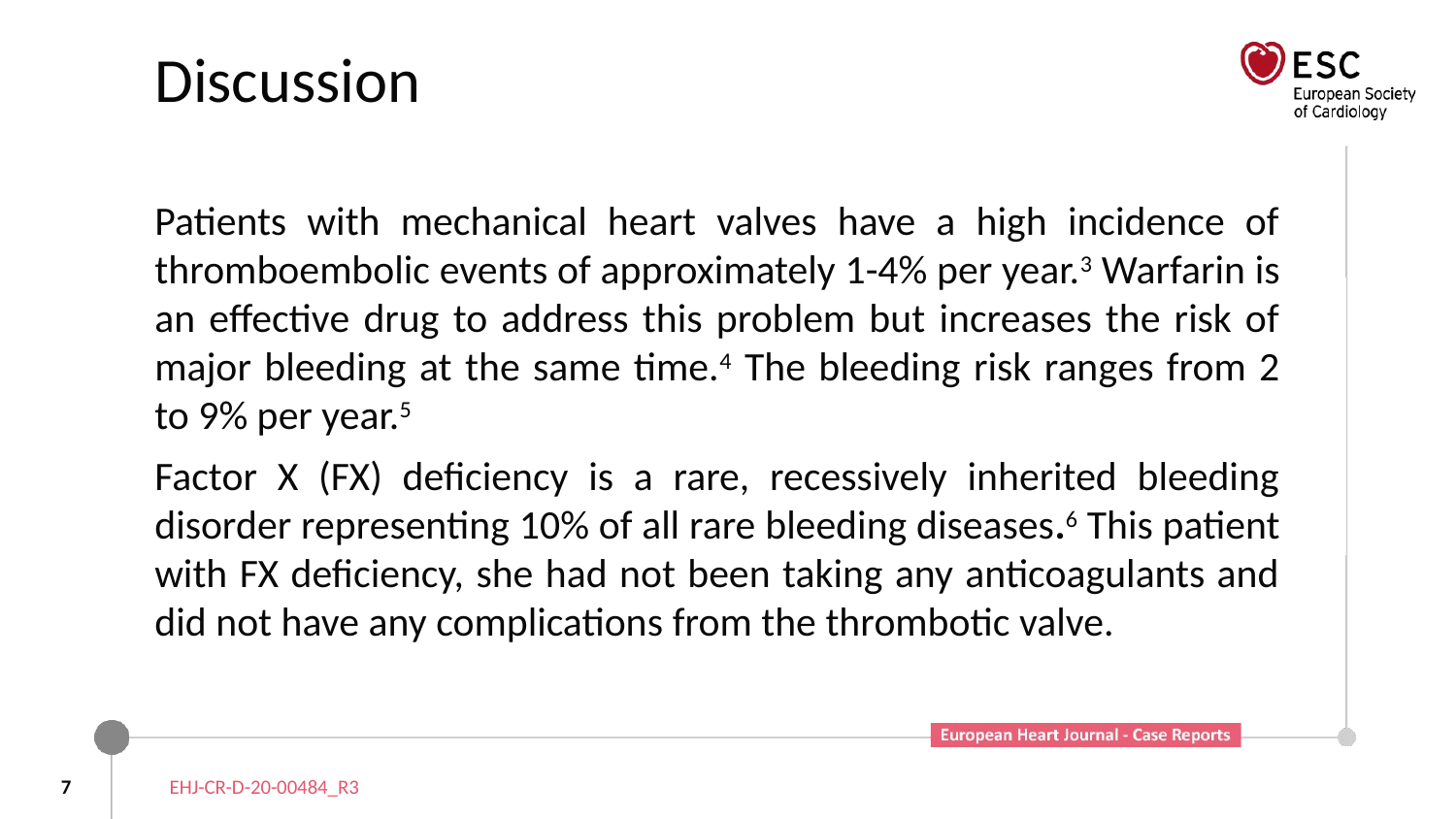

# Discussion
Patients with mechanical heart valves have a high incidence of thromboembolic events of approximately 1-4% per year.3 Warfarin is an effective drug to address this problem but increases the risk of major bleeding at the same time.4 The bleeding risk ranges from 2 to 9% per year.5
Factor X (FX) deficiency is a rare, recessively inherited bleeding disorder representing 10% of all rare bleeding diseases.6 This patient with FX deficiency, she had not been taking any anticoagulants and did not have any complications from the thrombotic valve.
7
EHJ-CR-D-20-00484_R3

## Slide 8
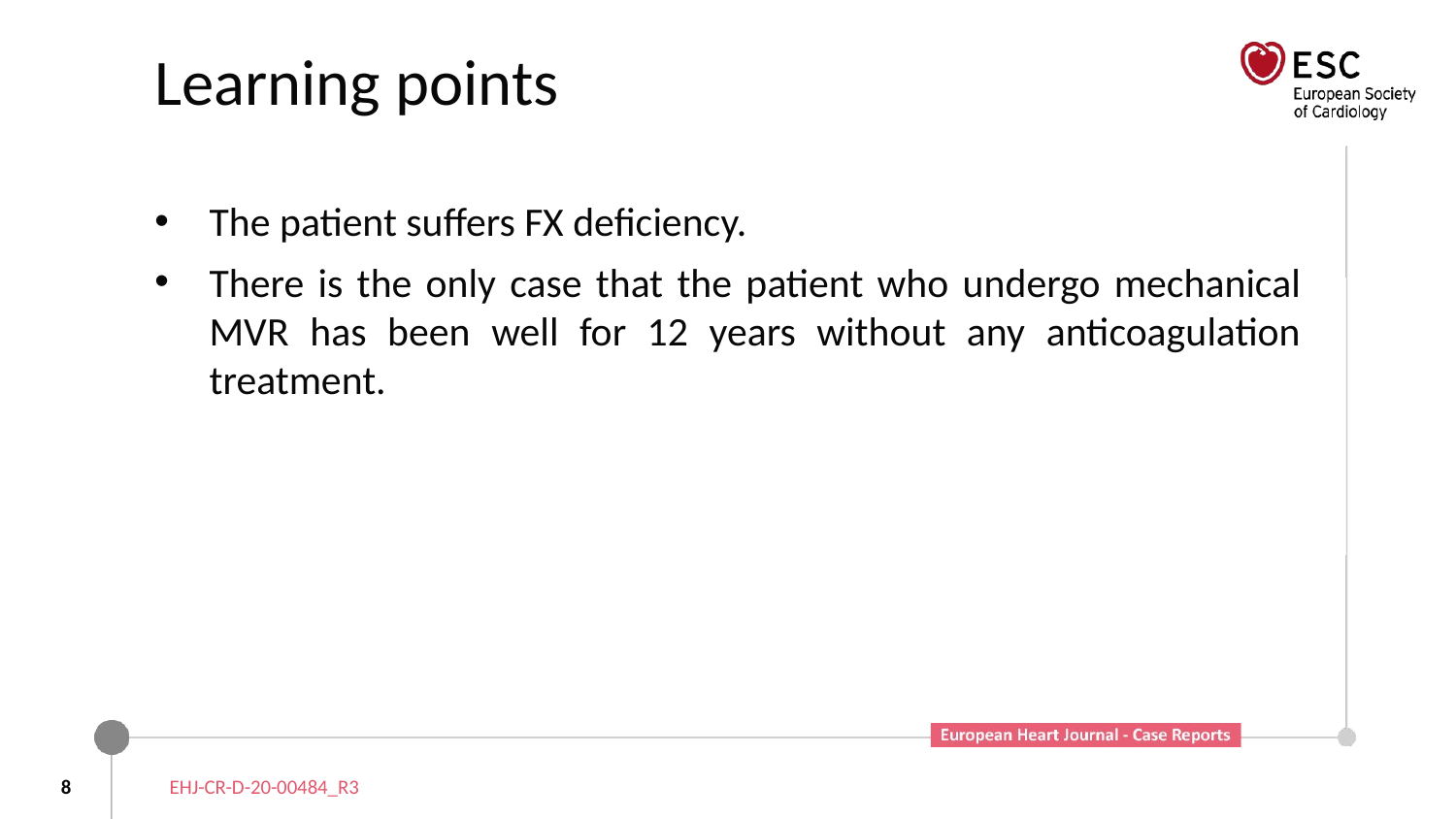

# Learning points
The patient suffers FX deficiency.
There is the only case that the patient who undergo mechanical MVR has been well for 12 years without any anticoagulation treatment.
8
EHJ-CR-D-20-00484_R3

## Slide 9
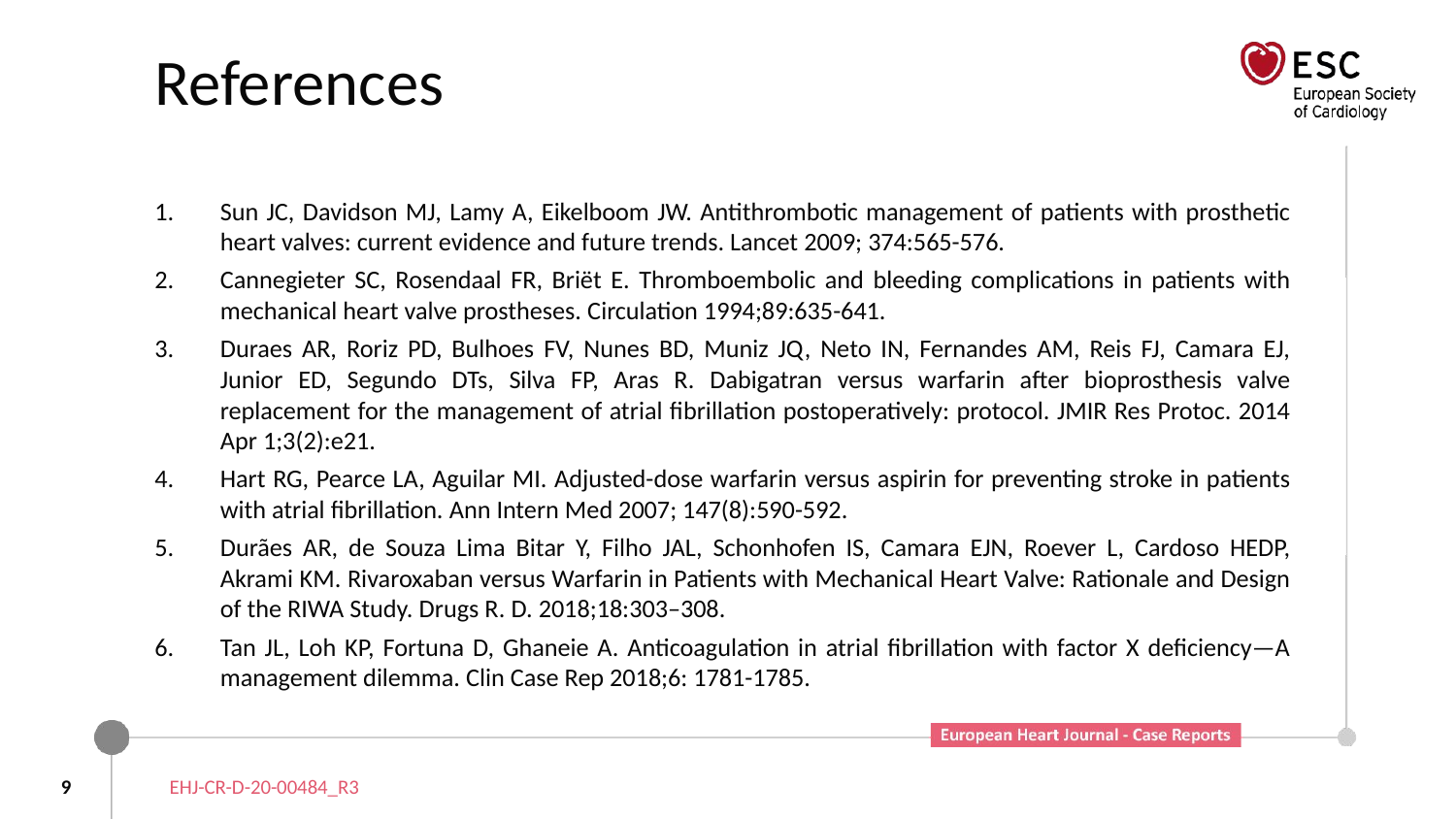

# References
Sun JC, Davidson MJ, Lamy A, Eikelboom JW. Antithrombotic management of patients with prosthetic heart valves: current evidence and future trends. Lancet 2009; 374:565-576.
Cannegieter SC, Rosendaal FR, Briët E. Thromboembolic and bleeding complications in patients with mechanical heart valve prostheses. Circulation 1994;89:635-641.
Duraes AR, Roriz PD, Bulhoes FV, Nunes BD, Muniz JQ, Neto IN, Fernandes AM, Reis FJ, Camara EJ, Junior ED, Segundo DTs, Silva FP, Aras R. Dabigatran versus warfarin after bioprosthesis valve replacement for the management of atrial fibrillation postoperatively: protocol. JMIR Res Protoc. 2014 Apr 1;3(2):e21.
Hart RG, Pearce LA, Aguilar MI. Adjusted-dose warfarin versus aspirin for preventing stroke in patients with atrial fibrillation. Ann Intern Med 2007; 147(8):590-592.
Durães AR, de Souza Lima Bitar Y, Filho JAL, Schonhofen IS, Camara EJN, Roever L, Cardoso HEDP, Akrami KM. Rivaroxaban versus Warfarin in Patients with Mechanical Heart Valve: Rationale and Design of the RIWA Study. Drugs R. D. 2018;18:303–308.
Tan JL, Loh KP, Fortuna D, Ghaneie A. Anticoagulation in atrial fibrillation with factor X deficiency—A management dilemma. Clin Case Rep 2018;6: 1781-1785.
9
EHJ-CR-D-20-00484_R3
